# Participation of calcium-permeable AMPA receptors in the regulation of epileptiform activity of hippocampal neurons

**DOI:** 10.3389/fnsyn.2024.1349984

**Published:** 2024-03-20

**Authors:** Valery Petrovich Zinchenko, Ilia Yu. Teplov, Artem Mikhailovich Kosenkov, Sergei Gennadievich Gaidin, Bakytzhan Kairatuly Kairat, Sultan Tuleukhanovich Tuleukhanov

**Affiliations:** ^1^Federal Research Center “Pushchino Scientific Center for Biological Research of the Russian Academy of Sciences”, Institute of Cell Biophysics of the Russian Academy of Sciences, Pushchino, Russia; ^2^Laboratory of Biophysics, Chronobiology and Biomedicine, Faculty of Biology and Biotechnology, Al-Farabi Kazakh National University, Almaty, Kazakhstan

**Keywords:** CP-AMPAR, Kv7, PDS, GABAergic neurons, epilepsy, epileptiform activity

## Abstract

**Introduction:**

Epileptiform activity is the most striking result of hyperexcitation of a group of neurons that can occur in different brain regions and then spread to other sites. Later it was shown that these rhythms have a cellular correlate *in vitro* called paroxysmal depolarization shift (PDS). In 13–15 DIV neuron-glial cell culture, inhibition of the GABA(A) receptors induces bursts of action potential in the form of clasters PDS and oscillations of intracellular Ca^2+^ concentration ([Ca^2+^]_i_). We demonstrate that GABAergic neurons expressing calcium-permeable AMPA receptors (CP-AMPARs) as well as Kv7-type potassium channels regulate hippocampal glutamatergic neurons’ excitability during epileptiform activity in culture.

**Methods:**

A combination of whole-cell patch-clamp in current clamp mode and calcium imaging microscopy was used to simultaneously register membrane potential and [Ca^2+^]_i_ level. To identify GABAergic cell cultures were fixed and stained with antibodies against glutamate decarboxylase GAD 65/67 and neuron-specific enolase (NSE) after vital [Ca^2+^]_i_ imaging.

**Results and discussion:**

It was shown that CP-AMPARs are involved in the regulation of the PDS clusters and [Ca^2+^]_i_ pulses accompanied them. Activation of CP-AMPARs of GABAergic neurons is thought to cause the release of GABA, which activates the GABA(B) receptors of other GABAergic interneurons. It is assumed that activation of these GABA(B) receptors leads to the release of beta-gamma subunits of Gi protein, which activate potassium channels, resulting in hyperpolarization and inhibition of these interneurons. The latter causes disinhibition of glutamatergic neurons, the targets of these interneurons. In turn, the CP-AMPAR antagonist, NASPM, has the opposite effect. Measurement of membrane potential in GABAergic neurons by the patch-clamp method in whole-cell configuration demonstrated that NASPM suppresses hyperpolarization in clusters and individual PDSs. It is believed that Kv7-type potassium channels are involved in the control of hyperpolarization during epileptiform activity. The blocker of Kv7 channels, XE 991, mimicked the effect of the CP-AMPARs antagonist on PDS clusters. Both drugs increased the duration of the PDS cluster. In turn, the Kv7 activator, retigabine, decreased the duration of the PDS cluster and Ca^2+^ pulse. In addition, retigabine led to deep posthyperpolarization at the end of the PDS cluster. The Kv7 channel is believed to be involved in the formation of PDS, as the channel blocker reduced the rate of hyperpolarization in the PDS almost three times. Thus, GABAergic neurons expressing CP-AMPARs, regulate the membrane potential of innervated glutamatergic neurons by modulating the activity of postsynaptic potassium channels of other GABAergic neurons.

## 1 Introduction

Epileptiform activity is the most striking result of hyperexcitation of a group of neurons that can occur in different brain regions and then spread to other sites. High-amplitude synchronous low-frequency rhythmic auto-oscillations (termed seizure precursors) are observed on the electroencephalogram in the periods between the seizures or before them (Hotka and Kubista, 2019). Similar hyperexcitation states were also shown in the case of an ischemic stroke, Alzheimer’s disease, Parkinson’s disease (Kuijlaars et al., 2016; Kubista et al., 2019), and during brain development (Mohajerani and Cherubini, 2006; Mohajerani et al., 2007). Although the seizure precursors can be registered in the first hours after the stroke, epilepsy attacks themselves are observed only a day to a week after (Holtkamp et al., 2017). Interestingly, in addition to neuronal network development, high-amplitude synchronous low-frequency rhythms occur after traumatic brain injury or brain concussion when connections between neurons are synchronously formed/restored (Bacci et al., 1999). It is believed that, in this case, such oscillations initiate the developmental process in a fully mature neuronal network. Shortly after these rhythms were discovered in a whole brain, it was shown that they have a cellular correlate *in vitro* called paroxysmal depolarization shift (PDS). PDSs were found in brain slices obtained from the epilepsy zone and cell cultures (Hotka and Kubista, 2019). In this way, epileptiform activity *in vitro* caused by attenuation of GABA(A)-receptor-mediated inhibition corresponds to interictal spikes *in vivo*. Thus, the problem of PDS initiation and regulation has become central to studies of hyperexcitation and its role in neuronal death and various neurodegenerative diseases. The difficulty in treating epilepsy and other hyperexcitation-accompanied CNS diseases is due to the complexity of the process, in which the expression of more than 1,000 genes is altered (Poduri and Lowenstein, 2011). On the one hand, the existence of a cellular correlate of the epileptiform activity simplifies the investigation of this phenomenon, but on the other hand, the multitude of components which contribution must be established to reveal the mechanism slows the progress in this area.

Epileptiform activity is characterized by complex (in terms of participants), nonlinear oscillations of membrane potential, hypersynchronization of numerous neurons, low frequency of the events, the high amplitude of depolarization, and oscillations of basal [Ca^2+^]_i_ (Ca^2+^ pulses) with complete pumping of Ca^2+^ from the cytoplasm in the period between pulses. Recent studies have revealed the involvement of many voltage- and ligand-gated ion channels in induction and maintaining action potential (AP) bursts and Ca^2+^ pulses (Zhang et al., 2019).

Calcium-permeable AMPA and kainite receptors (CP-AMPARs and CP-KARs) have recently been shown to play an important role in neuronal hyperexcitation. It has been shown that in the hippocampus of adult rats, CP-AMPARs are expressed mainly in GABAergic neurons, and these neurons account for about 35% of all GABAergic neurons (Zinchenko et al., 2020). Moreover GABAergic neurons expressing CP-AMPARs can innervate other GABAergic interneurons expressing CP-KARs and thus disinhibit glutamatergic neurons during epileptiform activity (Zinchenko et al., 2020, 2021). The CP-AMPA receptor antagonist NASPM reverses this effect. NASPM causes activation of interneurons and inhibition of glutamatergic neurons (Zinchenko et al., 2021).

The present work is devoted to further studying the mechanisms of epileptiform activity, particularly the role of CP-AMPARs and potassium channels in the regulation of PDS during epileptiform activity of neurons in culture. We have previously shown that during epileptiform activity, GABAergic neurons expressing CP-AMPARs and CP-KARs release GABA (Maiorov et al., 2021), which interacts with GABA(B) receptors, suppressing the hyperexcitation (Zinchenko et al., 2020). The effect may be due to the interaction of the βγ subunit of the Gi protein of GABA(B) receptor with certain potassium channels of target cells. The most likely candidate for this role is Kv7-type potassium channels. Finding out the role of CP-AMPARs and signal transduction pathways involving disinhibitory GABAergic interneurons in regulating epileptiform activity is a definite step in revealing the mechanisms of epileptiform activity. Data obtained open new targets for suppression of hyperexcitation in epilepsy and other neurodegenerative diseases.

## 2 Materials and methods

Experimental procedures were carried out according to the Council Directive 2010/63 EU of the European Parliament (September 22, 2010) on the protection of animals used for scientific purposes.

### 2.1 Preparation of hippocampal cell culture

Neuron-glial cell cultures were prepared as described previously (Gaidin et al., 2020, 2023; Maiorov et al., 2021; Laryushkin et al., 2023). Wistar pups (P0-2) were euthanized with deep-inhaled anesthesia and decapitated. Extracted brains were transferred into a plastic Petri dish (*d* = 60 mm) filled with cold Versene solution. The separated hippocampus was carefully minced with scissors and treated with 1% trypsin solution for 10 min at 37°C and with constant shaking. After the trypsinization, tissue was washed twice with a cold Neurobasal-A medium, and the tissue fragments were gently triturated using a 1 mL pipette tip. The obtained cell suspension was then centrifuged for 3 min at 2000 rpm, and the cell pellet was resuspended in cell culture medium consisted of Neurobasal-A medium supplemented with B27 (2%), 500 μM glutamine, and penicillin–streptomycin (1:100). The cell suspension was added into glass cylinders (internal diameter 6 mm, and the height 7 mm; 100 μL per cylinder) placed on the polyethyleneimine-coated cover glasses, and the Petri dishes were transferred to a CO_2_-incubator for 40 min for the cell attachment. Then, the cylinders were carefully removed, and 2 mL of the cell culture medium was added to each Petri dish. The cultures were grown at 37°C in a humidified atmosphere (humidity ≥90%) containing 5% CO_2_ and were used in experiments at 13–15 DIV (days *in vitro*).

### 2.2 Fluorescent [Ca^2+^]_i_ imaging and immunostaining

The changes in intracellular Ca^2+^ concentration ([Ca^2+^]_i_) were evaluated using fluorescent Ca^2+^-sensitive probe Fura-2 AM. All the imaging experiments were performed at 28°C in Hank’s balanced salt solution (HBSS) composed of (in mM): 136 NaCl, 3 KCl, 0.8 MgSO_4_, 1.25 KH_2_PO_4_, 0.35 Na_2_HPO_4_, 1.4 CaCl_2_, 10 glucose, and 10 HEPES; pH 7.35. Fura-2 stock solution (in dimethyl sulfoxide) was dissolved in HBSS to a final concentration of 3 μM. The cells were incubated with the probe for 40 min at 28°C and then washed thrice. The series of images were recorded using an inverted motorized fluorescent microscope Leica DMI 6000B (Leica Microsystems, Wetzlar, Germany) with a CCD camera Hamamatsu C9100 (Hamamatsu Photonics K.K., Hamamatsu City, Japan). Ratiometric measurements were performed using the set of filters, including the external filter wheel with excitation filters BP340/30 and BP387/15, and the internal FU-2 filter cube (dichroic mirror 72100bs, emission filter HQ 540/50 m; Leica Microsystems, Wetzlar, Germany). The images were analyzed with ImageJ (NIH, Bethesda, MD, United States) software, following the previously reported protocol (Maiorov et al., 2021). Changes in [Ca^2+^]_i_ are presented as 340/387 ratio for Fura-2. Short-term KCl applications were made before or after the experiments (not shown in panels) to identify neurons. The detailed protocol of the image analysis was described previously (Maiorov et al., 2021).

To identify GABAergic and non-GABAergic neurons (glutamatergic), cell cultures were fixed and stained with antibodies against glutamate decarboxylase GAD 65/67 and neuron-specific enolase (NSE) after vital [Ca^2+^]_i_ imaging. The detailed protocol of immunostaining was described previously (Maiorov et al., 2021; Laryushkin et al., 2023). A marker grid with a square side of approximately 3 mm was drawn on the bottom of the coverslips with the Fura-2-stained cell cultures before the vital fluorescent imaging experiments. After [Ca^2+^]_i_ measurements, the cells were photographed in the phase-contrast mode. Then, the cells were fixed with freshly prepared 4% paraformaldehyde solution for 20 min, washed with ice-cold phosphate-buffered saline, permeabilized with Triton X-100 solution, and stained with antibodies against glutamate decarboxylase GAD 65/67 (1:500) and neuron-specific enolase (NSE; 1:300). To block non-specific binding of the secondary antibodies, the cultures were incubated with 10% normal goat serum in PBS for 30 min. Primary antibodies were diluted in 1% normal goat serum with 0.1% Triton-X 100. The cultures were incubated with the primary antibodies for 12 h at 4°C, washed thrice with PBS, and incubated for 1 h with the secondary antibodies conjugated with the fluorescent probes. We used Goat anti-rabbit antibodies conjugated with Alexa Fluor 555 and Goat anti-mouse antibodies conjugated with Alexa Fluor 647. Antibody fluorescence and bright-filed images were recorded with Leica TCS SP5 inverted confocal microscope in the square of the marker grid where Fura-2 imaging was performed. The confocal images of antibody fluorescence and the images of Fura-2 fluorescence of the stained live cells were compared with ImageJ software.

### 2.3 Electrophysiological measurements

A combination of whole-cell patch-clamp in current clamp mode and calcium imaging microscopy was used to simultaneously register membrane potential and [Ca^2+^]_i_ level. A system for electrophysiological measurements is built into fluorescent station Axio Observer Z1 (Carl Zeiss, Germany), allowing simultaneous study of optical and electrophysiological characteristics of living cells (Gaidin et al., 2019; Kosenkov et al., 2019). All whole-cell recordings of membrane voltage were performed at 28°C in HBSS solution. The micropipettes were pulled from borosilicate glass capillaries with filament (Sutter Instrument, Navatto, CA, United States) using a vertical micropipette puller Narishige PC-100. The intrapipette solution was consisted of (in mM): 10 KCl, 125 K-gluconate, 1 MgCl_2_ × 6H_2_O, 0.25 EGTA, 10 HEPES, 2 Na_2_-ATP, 0.3 Mg-ATP, 0.3 Na-GTP, 10 Na_2_-phosphocreatine (pH 7.2; adjusted with 1 M KOH). Data were recorded and digitized with Axopatch 200B amplifier and a low-noise data acquisition system Axon DigiData 1440A digitizer (Molecular Devices, San Jose, CA, United States), respectively. The sampling rate was 10 kHz in all experiments.

### 2.4 Statistical and data analysis

ImageJ software (National Institutes of Health, Bethesda, Maryland, United States) was used for image analysis. Origin Pro 2021 version 9.8.0.200 was used for graph creation and analysis (OriginLab, Northampton, MA, United States). Electrophysiological data were analyzed using ClampFit 10 software (Molecular Devices, San Jose, CA, United States). The representative and mean kinetics (noted in the figure legends) are shown in most figures. In the case of electrophysiological experiments, the dots correspond to individual neurons (one neuron was analyzed in one cell culture preparation).

The fluorescence intensity was collected from the soma during the analysis of the time-lapse series. The view field shifts were corrected with the StackReg plugin of ImageJ. The mean background fluorescence for each series of the images was obtained by averaging the signals collected from 10 ROIs set on the areas of the culture without soma and processes of the cells, and the obtained mean background fluorescence kinetics (baselines) for each channel were subtracted from the corresponding signal of each analyzed cell. Two obtained datasets were used for the 340/387 ratio calculation.

The Shapiro–Wilk test (*p* > 0.05) was used to evaluate the normality of data distribution since the sample size was *n* < 15. Normality tests performed with Origin Pro showed that all datasets were normally distributed; therefore, we used t-tests. The differences were analyzed with paired t-tests (two-tailed) using GraphPad Prism 8 (GraphPad Software, San Diego, CA, United States). Significance levels are defined with a value of p less than 0.05. All experiments were performed using the cultures from at least 2–3 different animals. The number of independent repeats is marked as N or corresponds to the number of dots in the diagrams. This study was not pre-registered. No blinding or randomization was performed in this study. There were no exclusions, and no exclusion criteria were predetermined. No test for outliers was conducted.

### 2.5 Reagents

The reagents used in the experiments are listed below. (1) Sigma-Aldrich, Saint Louis, MO, United States: Poly(ethyleneimine) solution (Cat. no. P3143), penicillin–streptomycin (Cat. no. P4333), L-Glutamine (Cat. No G85402), Retigabine (Cat. No. SML0325), XE-991 (Cat. no. X2254). (2) Life Technologies, Grand Island, NY, United States: B-27 supplement (Cat. no. 17504044), Trypsin 2.5% (Cat. no. 15090046). (3) Molecular Probes, Eugene, OR, United States: Fura-2 AM (Cat. no. F1221). (4) Cayman Chemical, Ann Arbor, MI, United States: Bicuculline (Cat. no. 11727), 1-naphthylacetyl spermine (NASPM) (Cat. no. 18453). (5) Tocris Bioscience, Bristol, United Kingdom: ATPA (Cat. no. 11–071-0). (6) AppliChem, Darmstadt, Germany: EGTA (Cat. no. A-0878). (7) Dia-M, Moscow, Russian Federation: HEPES (Cat. no. 3350). (8) Paneco, Moscow, Russian Federation: Neurobasal-A medium.

## 3 Results

### 3.1 *In vitro* model

**Figure 1:**
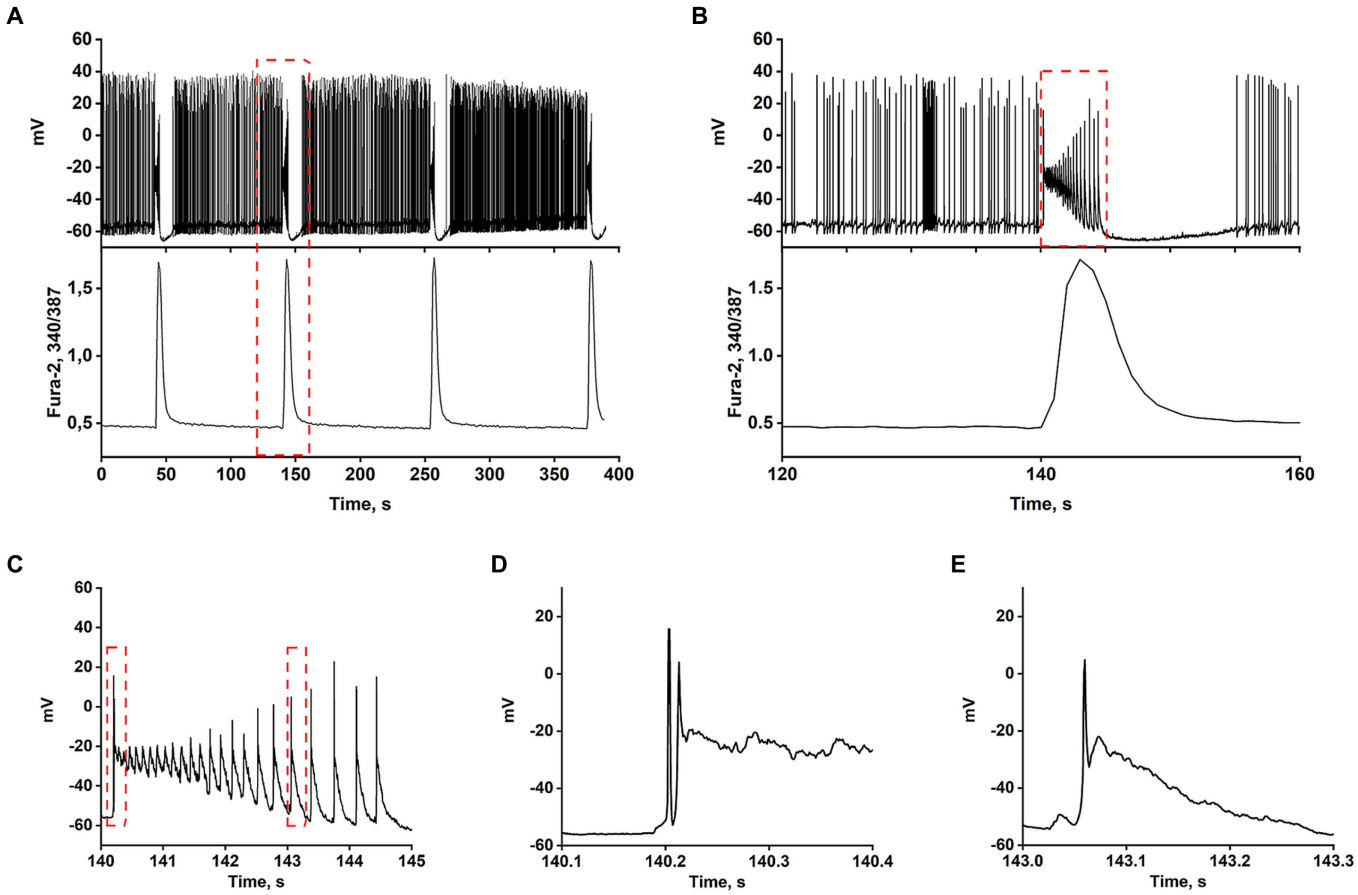
In the presence of bicuculline (10 μM), the spontaneous tonic activity of AP is periodically interrupted by burst activity accompanied by a Ca^2+^-pulse. **(A)** Changes in membrane voltage (top) and [Ca^2+^]_i_ (bottom) of a neuron. **(B)** Representative PDS cluster; changes in membrane potential (top panel) and [Ca^2+^]_i_ (bottom panel). **(C)** The magnified PDS cluster from Figure part label **В**. **(D)** The magnified leading edge of the cluster from Figure part label **C**. **(E)** One of the individual PDSs from Figure part label **C**. The AP is generated at the leading edge. The duration of the PDS is ~300 ms.

Most works have shown that the appearance of rhythmic epileptiform activity is related to deficits in GABAergic inhibition (Teplov et al., 2021). Consequently, GABA(A) receptor antagonists have become the simplest cellular model of epileptiform activity *in vitro* (Avoli et al., 2002; Salazar et al., 2003; Reddy and Kuruba, 2013; Khazipov, 2016; Gaidin et al., 2019). Synchronization of neuronal activity occurs in mature hippocampal cell culture in response to the blockade of GABA(A) receptors, which usually suppress inhibition by Cl^−^ inflow. Depolarization induced by GABA(A) receptor blockade removes the Mg^2+^ block from NMDAR, which increases the cluster amplitude and amplitude of Ca^2+^ pulses (Kosenkov et al., 2019). Figures 1A–E demonstrate that the spontaneous generation of action potentials (APs) by a pacemaker neuron in this mode is periodically interrupted by spontaneous burst activity called paroxysmal depolarizing shift (PDS) clusters. The GABA(A) receptor antagonist, bicuculline, increases the depolarization shift by an average of 15 mV, resulting in the generation of auto oscillations. Figure 1A shows that the membrane voltage fluctuations in the neuron are accompanied by [Ca^2+^]_i_ pulses only during the burst AP activity. In turn, Figure 1B demonstrates one of the PDS clusters. It is shown that APs disappear at the maximum of depolarization and gradually recover during repolarization. The amplitude of plateau depolarization (Figure 1D) tends to exceed the reactivation potential of Na^+^ channels so that all channels can be inactivated for some time. In this case, the inactivation potential (of all channels) is about −20 mV.

The AP burst ends with a phase of slow substantial posthyperpolarization (~10 mV below the threshold level), during which the impulse activity of the neuron stops. A phase of slow depolarization follows this, and when the potential reaches a threshold level, AP generates again.

Figure 1C shows that slow posthyperpolarization in the cluster is accompanied by an increase in AP amplitude, indicating the reactivation of the voltage-gated Na^+^ channels. At the leading edge of the cluster, high-frequency APs decaying in amplitude (due to inactivation of the voltage-gated Na^+^ channels) are generated (Figure 1D).

The time scaling of a single cluster is shown in Figures 1B,C. The cluster is mediated by the activity of two high-frequency oscillatory systems, one of which underlies the generation of action potentials, and the second one, slower (PDS), controls the frequency of these APs. Figure 1E demonstrates the time scaling of the single PDS. It is shown that the depolarization shift during PDS starts below the threshold potential for Na^+^ channels/AP generation, and is terminated in the area above the reactivation potential of inactivated Na^+^ channels. Therefore, a single AP is most often generated at the leading edge of PDS.

Thus, suppression of GABA(A)-receptor-mediated inhibition in the network causes synchronous high-amplitude low-frequency periodic AP bursts (PDS clusters).

### 3.2 Identification of neurons expressing calcium-permeable AMPA and KA receptors

GABAergic neurons mainly mediate inhibition in the neuronal network. However, numerous data demonstrate that activation of GABAergic neurons expressing CP-AMPARs often has an excitatory effect (Cohen et al., 2002; Köhling, 2002). As shown previously, this effect is due to their inhibition of other GABAergic neurons (Zinchenko et al., 2021) and disinhibition of downstream glutamate neurons.

We have previously reported the methods of vital identification of neurons expressing calcium-permeable AMPA and KA receptors (CP-AMPARs and CP-KARs) using Ca^2+^ imaging (Zinchenko et al., 2021; Gaidin et al., 2023). In order to visualize neurons expressing CP-AMPARs and CP-KARs in hippocampal neuron-glial cultures, we recorded changes in the cytosolic Ca^2+^ concentration ([Ca^2+^]_i_) induced by agonists and antagonists of these receptors. The domoic acid (DoA) was used as an agonist of both receptors (Hogberg and Bal-Price, 2011; Zinchenko et al., 2021). ATPA was used as a selective agonist of GluK1-containing CP-KARs (Maiorov et al., 2021). NASPM was used as a selective antagonist of CP-AMPARs (Twomey et al., 2018).

**Figure 2:**
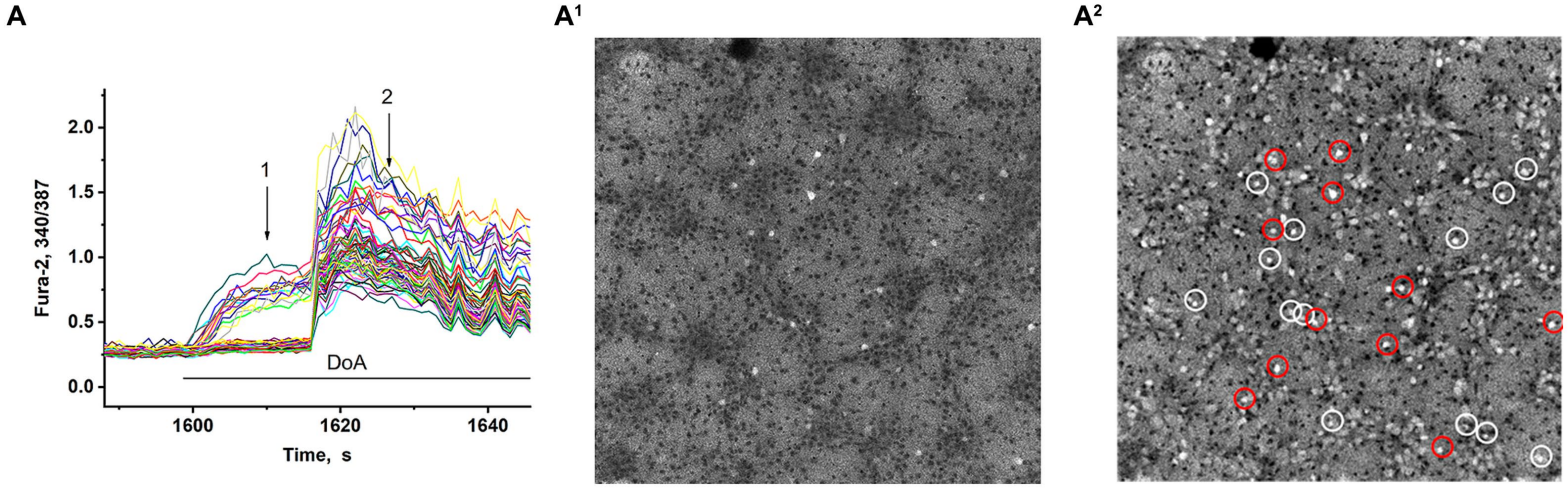
Visualization of neurons expressing CP-KARs and CP-AMRARs; **(A,A**^**1**^**)** DoA initially increases [Ca^2+^]_i_ in GABAergic neurons expressing CP-AMPA and CP-KA receptors. **(A**^**1**^**,A**^**2**^**)** Fluorescent images of cells stained with Fura-2 (340/387 ratio). Light cells are neurons excited during synchronous activity. The dark spots correspond to the cells with low [Ca^2+^]_i_. **(A**^**1**^**)** DoA-sensitive, fast-responding DoA neurons (expressing CP-KARs and CP-AMPARs) (registered at point 1). **(A**^**2**^**)** Light cells are all excited neurons with increased [Ca^2+^]_i_. The dark cells are astrocytes (registered at point 2). NASPM-sensitive neurons are circled in red. ATPA-sensitive neurons are circled in white.

In synchronized neuronal activity mode, we measured Ca^2+^ signals in all neurons in the field of view (150–200 neurons) (Figure 2). We have shown that DoA induced the rapid the [Ca^2+^]i increase in 15% neurons in hippocampal cell culture (Figures 2A,A^1^). All other neurons are excited with a delay of 12–15 s (Figures 2A,A^2^) (see also 16, 27). It has been shown that DoA induces a rapid Ca^2+^ response only in GABAergic neurons expressing CP-AMPA and CP-KA receptors (Maiorov et al., 2021; Gaidin et al., 2023). The selective antagonist of CP-AMPARs, NASPM, completely suppressed the rapid [Ca^2+^]_i_ increase in one subpopulation (red circles in Figure 2A^2^). In turn, a selective agonist of GluK1-containing CP-KAR, ATPA, increased the [Ca^2+^]_i_ only in a second subpopulation (light circles in Figure 2A^2^). Thus, calcium responses to ATPA and DoA allowed us to identify neurons expressing CP-KA and CP-AMPA receptors.

**Figure 3:**
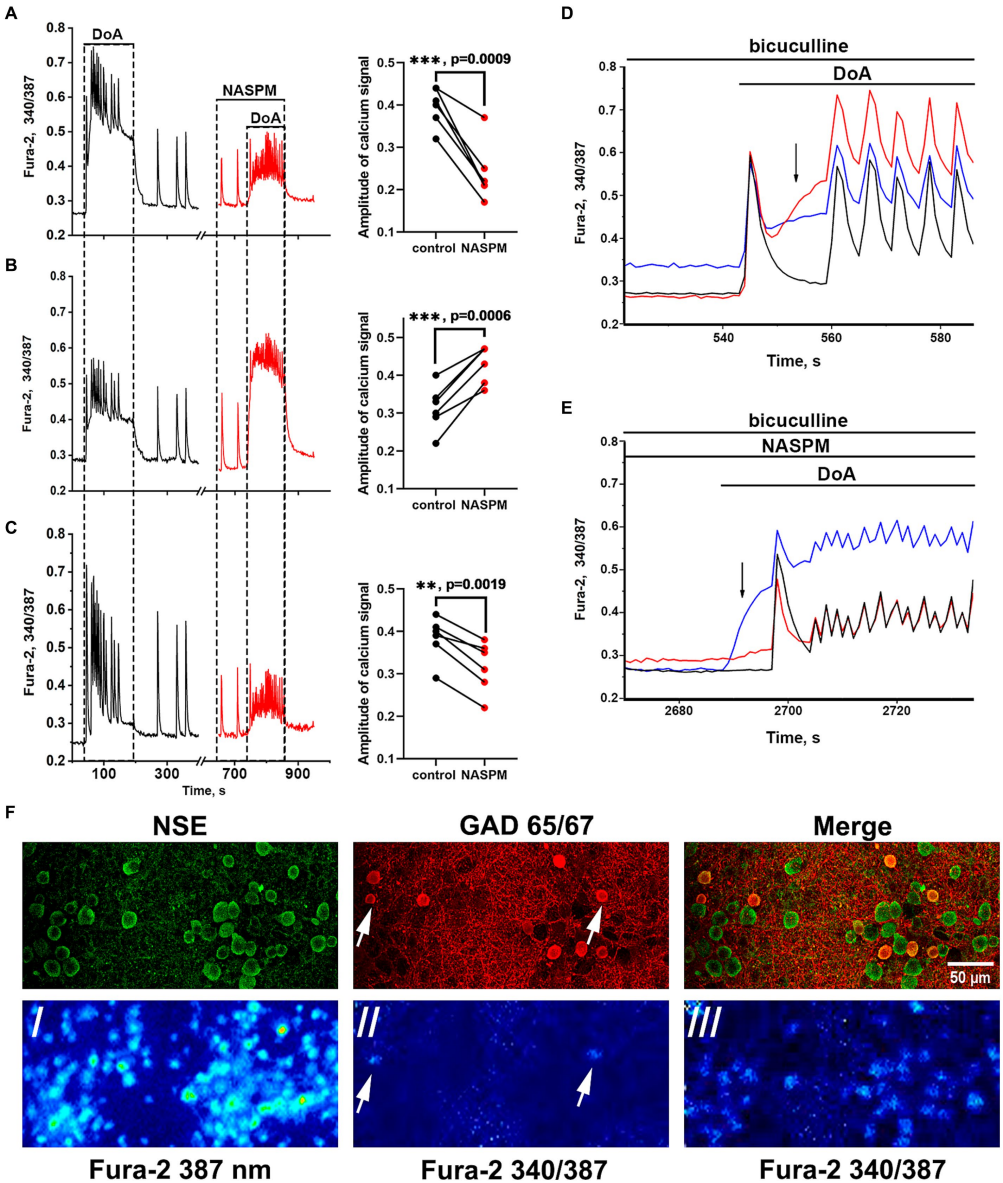
Effect of NASPM on [Ca^2+^]_i_ changes in neurons of the three subpopulations. Changes in [Ca^2+^]_i_ during spontaneous synchronous neuronal activity and in response to DoA (300 nM) application in control (black curves, left part) and in the presence of NASPM (50 μM) (red curves) in three subpopulations of neurons in the representative experiment: **(A)** GABAergic neurons expressing CP-AMPAR (10 cells), **(B)** GABAergic interneurons (7 cells) and **(C)** glutamatergic neurons (31 cells). The average traces are shown on the graphs. The diagrams presented on the right in panels A-C demonstrate the changes in the amplitude of the Ca^2+^ response of neurons in control and in the presence of NASPM; paired t-test. Each dot corresponds to the mean amplitude of each subpopulation in an individual experiment. **(D,E)** The averaged Ca^2+^ signals of neurons from each family. Blue curves correspond to NASPM-insensitive neurons expressing CP-KAR, red curves correspond to neurons expressing CP-AMPAR, and black curves correspond to glutamatergic neurons. The response to DoA in control **(D)** and in the presence of NASPM **(E)**. **(F)** Images of Fura-2 fluorescence and distribution of the antibodies against NSE and GAD 65/67 in the representative area of cell culture, which averaged Ca^2+^ responses are shown in panels D and E./- the image of Fura-2 fluorescence upon 387 nm excitation demonstrating all cells in the view field;/ and /// - 340/387 ratio images showing the elevated [Ca^2+^]_i_ level in representative GABAergic neurons (white arrows) from both families at the moment marked with black arrow in panel **D** (//) and the [Ca^2+^]_i_ increase in all neurons during subsequent DoA-induced oscillations (///).

Membrane potential was measured in neurons by the patch-clamp method in whole-cell configuration. In all experiments, neurons from both subpopulations were positively stained with antibodies against glutamate decarboxylase 65/67 (Figure 3F) that correlated with our previous study demonstrating the presence of large amount of GABA in neurons containing CP-KARs and CP-AMPARs (Gaidin et al., 2023). This approach makes it possible to evaluate changes in [Ca^2+^]_i_ in these two subsets of GAD65/67-positive neurons and GAD65/67-negative neurons. Figure 3 shows [Ca^2+^]_i_ changes during spontaneous synchronous activity and in response to the DoA application in control and in the presence of NASPM in these three groups of neurons: (A) - in GABAergic neurons expressing CP-AMPARs, (B) - in GABAergic neurons expressing CP-KARs, and (C) - in glutamatergic neurons; panels D and E demonstrate the magnified initial parts of averaged DoA-induced Ca^2+^ signals in neurons from each family. In control, DoA was shown to initially increases [Ca^2+^]_i_ only in GABAergic neurons expressing CP-AMPARs or CP-KARs (Figure 3D). In glutamatergic neurons, the excitation signal is recorded with a delay. NASPM suppresses the initial [Ca^2+^]_i_ rise in GABAergic neurons expressing CP-AMPARs and suppresses the amplitude of the DoA-induced Ca^2+^ signal in glutamatergic neurons (Figures 3C,E). NASPM increases the amplitude of the DoA-induced Ca^2+^ signal only in the group of GABAergic neurons expressing CP-KARs (Figures 3B,E), assuming that neurons expressing CP-AMPARs innervate GABAergic neurons expressing CP-KARs. Notably, ATPA always increases [Ca^2+^]_i_ only in this group of GABAergic neurons.

### 3.3 Involvement of CP-AMPARs of GABAergic neurons in the regulation of epileptiform activity

#### 3.3.1 The effect of CP-AMPARs antagonist

The main experimental task was to show that the mechanism of glutamatergic neurons excitation during activation of CP-AMPAR GABAergic neurons, is realized during epileptiform activity of hippocampal neurons. A cell model in the mode of synchronous epileptiform activity was used to determine which parameters of the pre-epileptic rhythm induced by GABA(A)-receptor inhibition change alongside the changes in CP-AMPARs activity.

**Figure 4:**
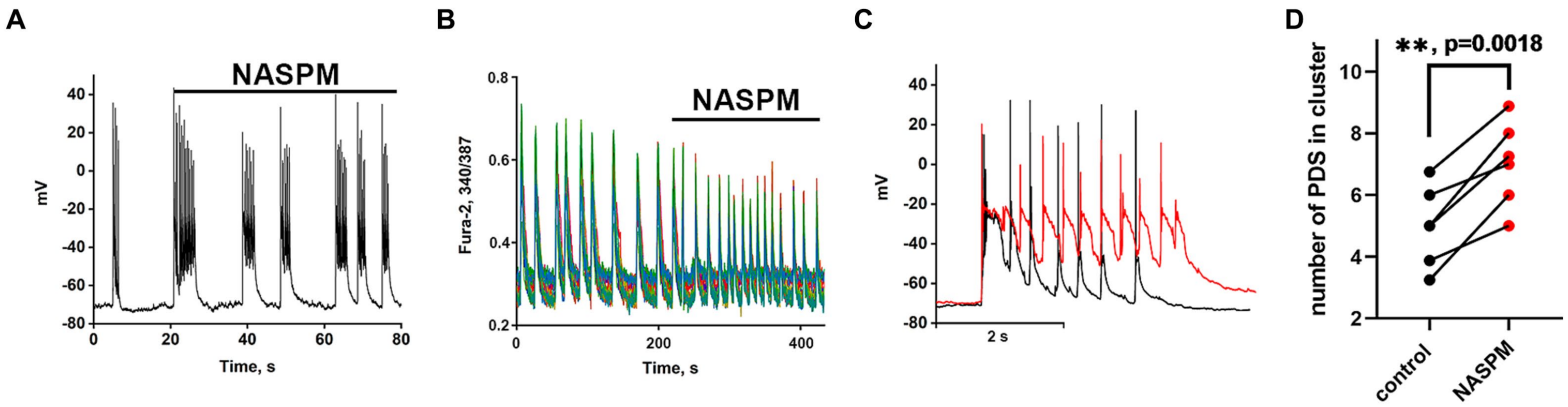
The effect of NASPM on PDS clusters and Ca^2+^ oscillations during bicuculline-induced epileptiform activity. **(A)** Changes in burst activity of the GABAergic interneuron innervated by GABAergic neurons containing CP-AMPARs. **(B)** Changes in the pattern of synchronous calcium oscillations in glutamatergic neurons in the presence of NASPM (50 μM) *N* = 4. **(C)** Changes in the membrane potential of a neuron in a cluster in the mode of epileptiform activity induced by a GABA **(A)** receptor antagonist (bicuculline 10 μM). Representative PDS clusters in control (black curve) and in the presence of NASPM (red curve). **(D)** Diagram showing the number of individual PDSs in the cluster in control and after NASPM application; paired t-test. Each dot corresponds to the mean number of the PDSs in individual neurons before (black dots) and after (red dots) NASPM application.

Recordings of the membrane potential of the GABAergic neuron innervated by GABAergic containing CP-AMPARs during bicuculline-induced epileptiform activity (A) and the changes in Ca^2+^ pulses (B) in glutamatergic neurons are shown in Figure 4. In this case, the membrane potential oscillations appear as AP bursts occurring against the background of periodic large depolarizing shifts. The PDS clusters in control and in the presence of NASPM are superimposed in Figure 4C. Before the application of the CP-AMPARs antagonist, NASPM, the amplitude of the PDS cluster decreased after the depolarizing shift (Figure 4C black curve). NASPM interrupted this process and restored the amplitude to maximum (Figure 4C red curve). Rapid hyperpolarization in the control is also observed in each PDS. We analyzed 10 clusters in each group. The rate of PDS hyperpolarization in the cluster was 475 ± 120 mV/s in control. In the presence of NASPM the rate decreased to 80 ± 7 mV/s. The cluster maximum amplitude in the presence of NASPM was −25 ± 2 mV in the third and fourth PDS. In control, the maximum amplitude was −25 ± 2 mV and decreased to −47 mV after 4^th^ PDS (Figure 4C). The maximum depolarization shift amplitude was 23 mV in control and 26 mV in the presence of NASPM. Thus, NASPM had no effect on the cluster amplitude but completely removed the hyperpolarization phase in the cluster and slowed it down in the PDS (Figure 4C, red curve). The selective effect of NASPM only on the hyperpolarization phase of both cluster and PDS proves that the depolarization shift and hyperpolarization are formed by different ion channels, confirming the independence of these processes. In other words, the depolarization shift does not depend on the potential regulated by potassium channels.

The experiments showed that CP-AMPARs are involved in the regulation of PDS patterns. However, only hyperpolarization is regulated, but not the depolarization shift.

Thus, GABAergic neurons expressing CP-AMPARs can be used for selective regulation of the potassium channels that control the duration of PDS clusters and Ca^2+^ pulses in glutamatergic neurons.

Analysis of the participation of GABAergic neurons expressing CP-AMPARs in the regulation of potassium channels during epileptiform neuronal activity allows us not only to establish the pathway of signal transduction from these receptors to potassium channels but also helps to determine the nature of the channels involved in the generation of PDS clusters.

A low-threshold and long-lived Kv7-type potassium channel localized mainly in the axon hillock region may determine the duration of the PDS cluster because it operates longer than other voltage-gated channels that are already inactivated at these times (Brown and Passmore, 2009). The channel is controlled by potential and chemical ligands, such as PIP2, βγ subunits of G proteins, and Ca^2+^-CaM (Fanger et al., 1999; Schumacher et al., 2001; Adelman, 2016). To show that Kv7 channels are involved in PDS regulation, we investigated the effects of the direct blocker and activator of this channel on PDS clusters.

### 3.4 Effect of Kv7 channel activator on PDS clusters

**Figure 5:**
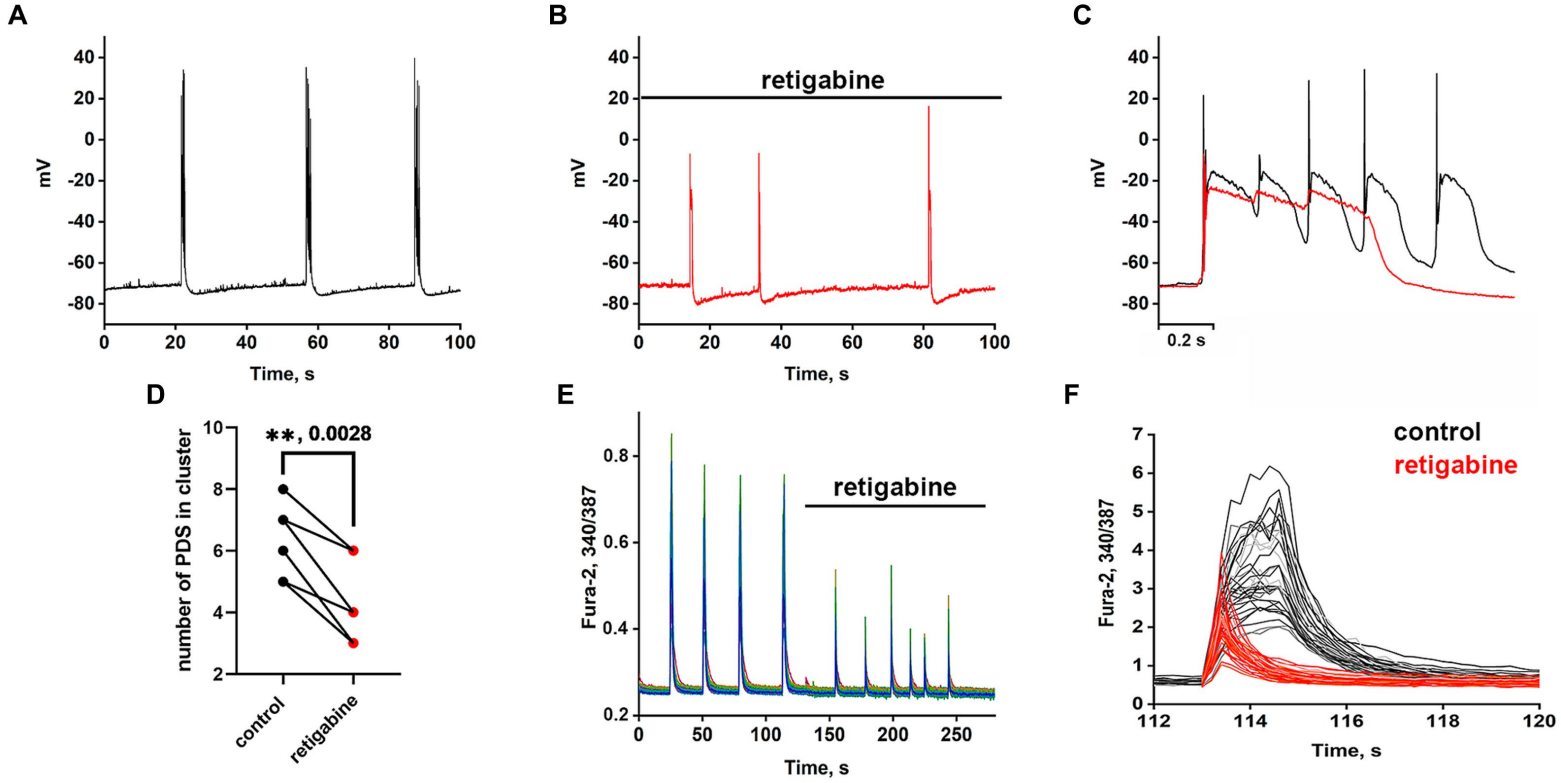
Periodic PDS clusters in glutamatergic neurons during bicuculline-induced epileptiform activity in control **(A)** and after retigabine (2.5 μM) application **(B)**. **(C)** Enlarged and superimposed PDS clusters in control (black curve) and in the presence of retigabine (red curve). **(D)** Diagram showing the number of individual PDSs in the cluster in control and after retigabine application; paired t-test. Each dot corresponds to the mean number of the PDSs in individual neurons before (black dots) and after (red dots) retigabine application. **(E)** Spontaneous synchronized epileptiform Ca^2+^ pulses in control and after retigabine addition. The half-width of the pulses decreases from 2 s to 0.8 s in the presence of retigabine; *N* = 4. **(F)** Ca^2+^ pulses in control and in the presence of retigabine. Decrease in Ca^2+^ pulse duration after the addition of retigabine (from Figure part label **E**). Black and red curves are signals from the same cells, before (black) and after the addition of retigabine (red). All neurons are glutamatergic.

The Kv7 activator retigabine is currently being tested as an anticonvulsant, analgesic, and anti-inflammatory agent (Miceli et al., 2008; Gaetano, 2012). Figures 5A–C show the bursting activity of a glutamatergic neuron in the mode of periodic PDS cluster generation induced by bicuculline in control (A) and after the application of 2.5 μM retigabine (B). In the control, the cluster consisted of 5–6 PDS, while the number of PDSs in the cluster decreased to three after the activator application. Figure 5C compares PDS clusters in control and in the presence of retigabine. Retigabine has been shown to reduce the cluster duration and increase the posthyperpolarization after the PDS cluster. The duration of the clusters decreases from 1.2 ± 0.2 s to 0.5 ± 0.2 s in the presence of retigabine. The average posthyperpolarization was 4.5 ± 0.3 mV in control and 8.6 ± 0.6 mV in the presence of retigabine, indicating a twofold increase. In control, we observed 5–6 individual PDSs in the cluster, whereas in the presence of the activator, the cluster consisted of 2–3 PDSs.

As can be seen in Figure 5, the hyperpolarization phase is completely absent in the PDS structure, indicating that Kv7 channels are involved in the hyperpolarization processes of the cluster and PDS. Apparently, activation of the channel hyperpolarizing PDS requires a chain of events, including reactivation of Na^+^ and K^+^ channels and generation of APs, which remain inactivated in the presence of retigabine. The amplitude of the cluster is also slightly reduced. Ca^2+^ pulses in the mode of epileptiform activity in control (after the addition of bicuculline) and after retigabine application are shown in Figures 5E,F. It is demonstrated that the activator of Kv7 channel decreases the duration of Ca^2+^ pulses and decreases the amplitude of the signal. Half width of Ca^2+^ pulses averaged over 150 glutamatergic neurons in 4 experiments decreased from 1.6 ± 0.2 s in the control to 0.75 ± 0.2 s in the presence of the activator. The amplitude of the Ca^2+^ pulse in each cell decreased by 58 ± 6% on average. Thus, it can be concluded that Kv7 channels participate at least in the cluster and Ca^2+^ signal termination and reduce their duration.

### 3.5 The effect of the Kv7 blocker on PDS clusters

**Figure 6:**
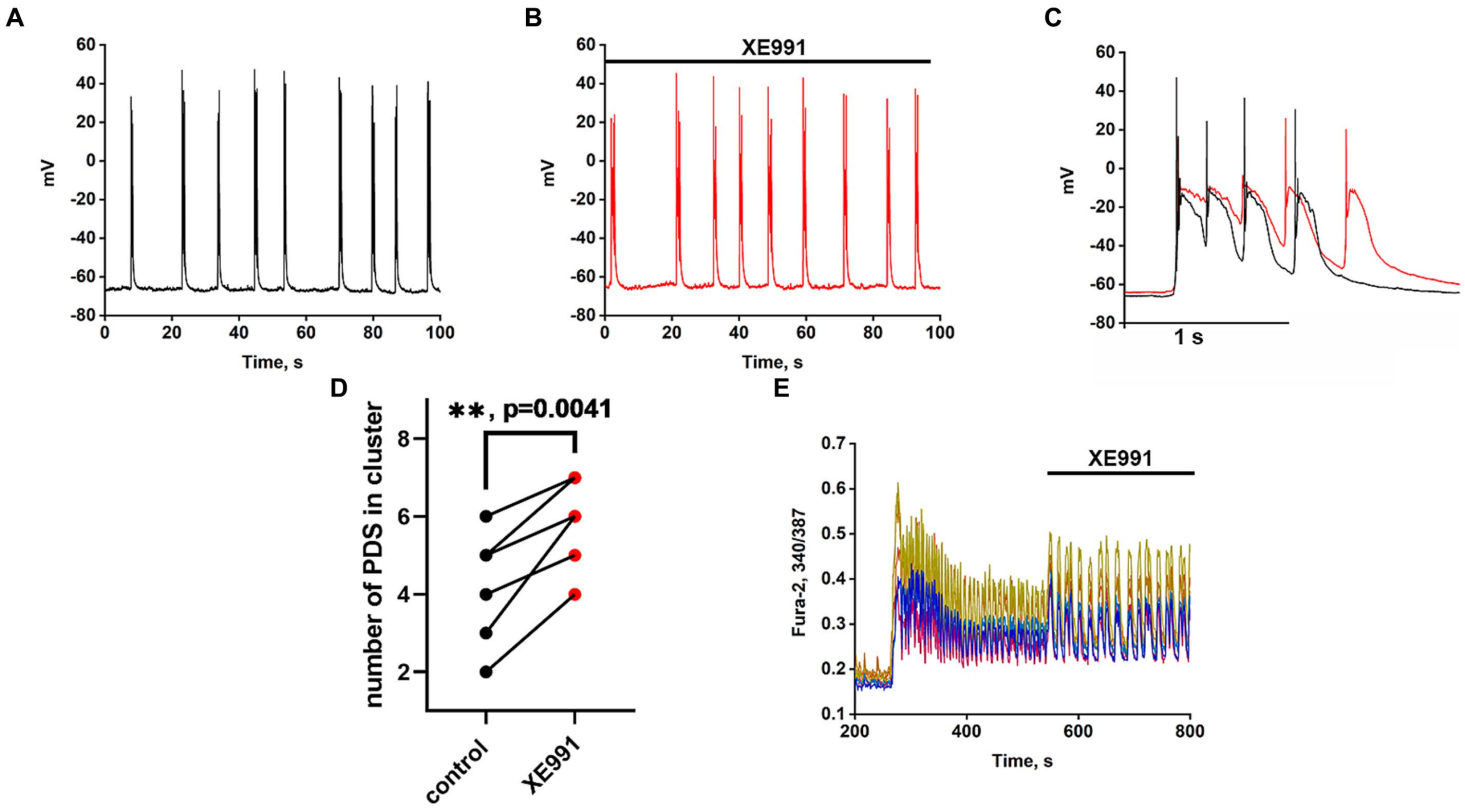
Effect of the Kv7 blocker XE991 (10 μM). **(A)** Periodic AP bursts (PDS clusters) generated by a glutamatergic neuron in the presence of bicuculline in control. **(B)** Periodic AP bursts (PDS clusters) generated by the same neuron in the presence of the Kv7 blocker XE991; *N* = 4. **(C)** Comparison of two clusters, in control (black curve) and in the presence of the Kv7 blocker XE991 (red curve). In the control, the rate of hyperpolarization in the first PDS is 125 ± 2 mV/s, which decreases to 44 ± 2 mV/s in the presence of the blocker. PDS clusters were taken from Figures part labels **A,B**. **(D)** Diagram showing the number of individual PDSs in the cluster in control and after XE991 application; paired t-test. Each dot corresponds to the mean number of the PDSs in individual neurons before (black dots) and after (red dots) XE991 application. **(E)** Changes in [Ca^2+^]_i_ in 8 random neurons in response to 8 mM NH4Cl and 20 μM XE991. The Kv7 channel blocker XE991 increases the duration of Ca^2+^ pulses (from 6 to 10 s). *N* = 3.

Recordings of the neuronal membrane potential during bicuculline-induced epileptiform activity in control (Figure 6A) and in the presence of the Kv7 channel blocker 4-pyridinyl methyl-9 (10H)-anthracenone (XE991) (Figure 6B) are shown in Figure 6. Comparison of PDS clusters in experiment and control (Figure 6C) indicates that XE991 increases cluster duration and decreases the rate of hyperpolarization. The figure shows that the hyperpolarization rate is not constant in each PDS and increases in each subsequent PDS in the cluster. Therefore, we compared the hyperpolarization rates only in the first PDSs. The hyperpolarization rate in the presence of XE991decreases almost 3-fold from 125 ± 3 to 44 ± 2 mV/s.

PDS clusters in the control consist of 3–4 PDSs (Figure 6C), while the number of PDSs in the cluster increased to 4–5 after the XE991 application. The increase in PDS cluster duration occurs due to the appearance of additional PDSs (Figure 6C red curves). The blocker does not affect the PDS amplitude, i.e., it does not act on the depolarization shift.

Figure 6E shows that the Kv7 channel blocker ХЕ991 increases the duration of Ca^2+^ pulses. The half-width of the average Ca^2+^ pulse from 8 neurons and 10 pulses in each neuron is 4.8 ± 0,3 s in control and 8.0 ± 0.3 s in the presence of XE991, which correlates with an increase in PDS cluster duration (Figure 6C). The mean duration for 21 control clusters was 0.7 ± 0.2 s and it increased in 14 clusters in the presence of XE991 to 1.0 ± 0.2 s.

The results showed that the duration of the PDS cluster is discrete (varies by an integer number of PDS) and changes in the control by 1 PDS, indicating the participation of a stochastic process in the induction of the cluster. We found that Kv7 channels contribute to PDS cluster formation. The Kv7 blocker XE991 increases, while the Kv7 activator retigabine, on the contrary, decreases the duration of the PDS cluster. Both drugs mainly affect hyperpolarization and have little effect on the depolarization shift. Moreover, the channel activator, as suggested, enhances the deep hyperpolarization at the end of the PDS cluster, which is required to reactivate the other inactivated Na^+^ and Ca^2+^ channels (Gaetano, 2012).

Thus, we have shown that CP-AMPARs and Kv7-type potassium channels regulate the PDS cluster and Ca^2+^ pulse during epileptiform neuronal network activity.

**Figure 7:**
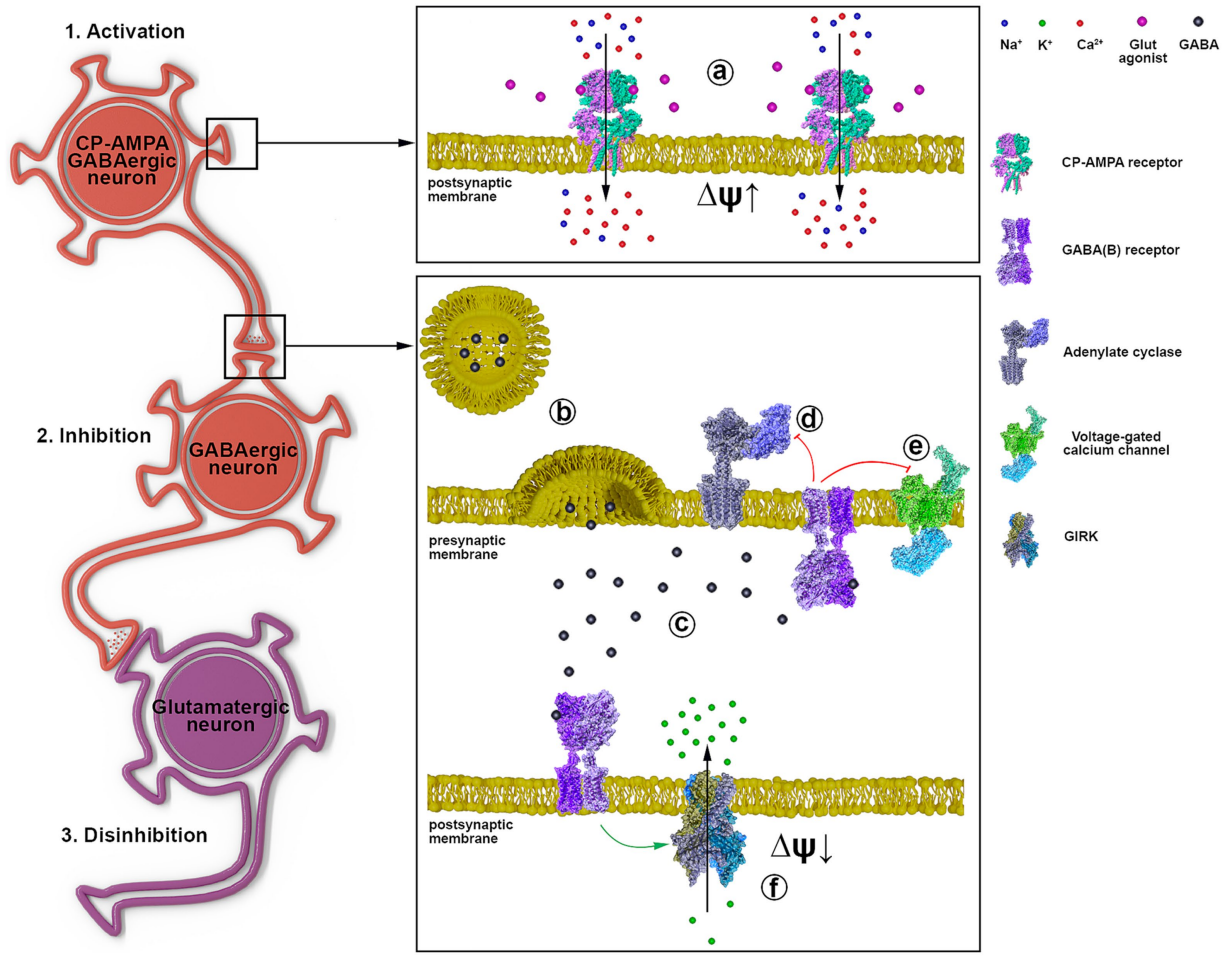
The representative scheme illustrates the signal transduction mechanism involving GABAergic neurons containing CP-AMPARs. 1 a - activation of CP-AMPA receptors of the GABAergic neuron by glutamate or depolarization, leading to [Ca^2+^]_i_ increase; 2 b - GABA release from the presynaptic terminal; c - binding of GABA to postsynaptic and presynaptic GABA(B) receptors; d - Gα subunit inhibits adenylyl cyclase; e - Gβγ subunit inhibits voltage-gated calcium channels; f - Gβγ subunit activates GIRK, hyperpolarizing the postsynaptic membrane; 3. Disinhibition of the glutamatergic neuron due to GABAergic neuron (Kuijlaars et al., 2016) inhibition.

Based on the obtained data and literature data, Figure 7 shows the signal transduction from a GABAergic neuron (Hotka and Kubista, 2019) expressing CP-AMPA receptors through another GABAergic interneuron (Kuijlaars et al., 2016) innervating a glutamatergic neuron (Kubista et al., 2019). Excitation of the GABAergic neuron (1, a) results in increased GABA release (b) and its interaction with GABA(A) (not shown) and GABA(B) receptors (c). GABA(B) receptors are localized both in the presynaptic and postsynaptic membranes (f). When GABA(B)R is activated, the βγ subunit of the G_i_ protein is released, activating postsynaptic K^+^ channels (f) and inhibiting presynaptic Ca^2+^ channels (e). Inhibition of cAMP synthesis by the αi subunit is similar (d), but the effect lasts longer. Opening of K^+^ channels hyperpolarizes and inhibits interneurons (Kuijlaars et al., 2016), and disinhibits glutamatergic neurons (Kubista et al., 2019). Closure of Ca^2+^ channels on the presynaptic membrane of GABAergic neurons (Hotka and Kubista, 2019) terminates/interrupts signaling by inhibiting Ca^2+^-dependent GABA release. Thus, activation of GABAergic neurons expressing CP-AMPA receptors may underlie the hyperexcitation of glutamatergic neurons in epilepsy. The scheme shows a possible mechanism of CP-AMPAR participation in the selective control of the activity of an individual neuronal population.

## 4 Discussion

The role of low-threshold Ca^2+^ and potassium channels in initiating AP bursting activity and regulating PDS duration has been shown previously (Teplov et al., 2021). In the present study, we show for the first time that potassium Kv7 channels control the duration of PDS clusters and activate posthyperpolarization in glutamatergic neurons during epileptiform activity in neuronal network *in vitro*.

CP-AMPARs of GABAergic neurons also regulate the duration of PDS clusters. The regulation appears to be due to the involvement of GABA(B) receptors, βγ subunits of Gi proteins, and activation of potassium channels. Kv7 channels appear to be involved in signal transduction from GABAergic neurons expressing CP-AMPAR to glutamatergic neurons.

Due to high affinity for glutamate and high excitability (Zinchenko et al., 2017; Olde Engberink et al., 2018; Goutierre et al., 2019), CP-AMPARs can also be classified as low-threshold receptors/channels, like T-type Ca^2+^ and Kv7 potassium channels. Thus, their involvement in PDS regulation could be predicted, but given their different localization and dynamics, a specific outcome (excitation or inhibition) is difficult to predict. Since Figure 3 shows that the CP-AMPAR antagonist activates only GABAergic neurons expressing CP-KARs and suppresses the activity of GABAergic neurons expressing CP-AMPARs and glutamatergic neurons, we assume that activation of GABAergic neurons expressing CP-AMPARs would disinhibit a large family of glutamatergic neurons by suppressing the activity of other inhibitory interneurons and enhance excitation. In this case, in the mode of epileptiform activity caused by the weakening of GABA-dependent inhibition (bicuculline is present in the extracellular medium), the regulation of excitation will be realized via GABA(B)-receptor-dependent activation/inhibition of potassium channels (presumably of the Kv7 type). The participation of GABA(B)-receptor in this process has been shown previously (Zinchenko et al., 2020).

It was shown earlier that NASPM selectively suppresses [Ca^2+^]_i_ increase and excitation in a specific population of GABAergic neurons (the first neuron in Figure 7) and simultaneously activates another population of inhibitory neurons (the second neuron in Figure 7). Measurements of the membrane potential in the neurons of this population, performed in the present study, showed that the activation of the neurons is caused by the inhibition of a potassium channel (presumably of the Kv7 type). It is suggested that channel activity is reduced due to attenuated dissociation of the βγ-subunits of the Gi protein coupled with the GABA(B) receptor. It has been previously shown that the βγ-subunit of Gi protein directly enhances Kv7 activity by increasing the channel’s sensitivity to PIP2 (Mori and Inoue, 2014; Povstyan et al., 2017).

The fact that Kv7 channels control cluster duration and post-cluster deep hyperpolarization is not unexpected, as they are known to be inactivated more slowly than other channels (Brown and Passmore, 2009).

The effects of direct blockers and activators of Kv7 confirmed the channel’s involvement in the regulation of PDS cluster duration and post-cluster repolarization. This critical fact opens up a new way to find drugs that selectively regulate these processes (amplitude and duration of PDS clusters).

It was previously shown that GABAergic neurons expressing CP-AMPARs are characterized by high maximal amplitude of cluster PDS depolarization and relatively strong subsequent hyperpolarization (Kosenkov et al., 2019; Gaidin et al., 2023). The hyperpolarization has been suggested to result from the activation of calcium-dependent potassium channels (Timofeev et al., 2004; Hotka and Kubista, 2019). However, the present work shows that NASPM abolishes hyperpolarization of the PDS cluster, apparently by inhibiting some other GIRK potassium channels (presumably Kv7). This fact does not negate the paradigm of the Ca^2+^-dependent regulation of cluster duration since Kv7 activity is also regulated by the Ca^2+^-calmodulin complex (Adelman, 2016).

The effect of direct activators and blockers of Kv7 channels is less selective than that of NASPM because they act on all Kv7 channels localized in different types of neurons (Shah et al., 2002). If Kv7 are localized in pyramidal neurons, their blocking by XE991 depolarizes the cell, enhances spike post-depolarization, and induces the burst-firing activity of neurons (Yue and Yaari, 2004). In contrast, NASPM alters Kv7 channel activity only in GABAergic neurons. Apparently, this non-selective action of the Kv7 channel ligands is associated with an increase in the amplitude of the Ca^2+^ signal in response to the Kv7 channel blocker and a decrease in the cluster duration and Ca^2+^ signal amplitude upon the Kv7 channel activation (Figure 5F).

Thus, the signal transduction pathway from GABAergic neurons expressing CP-AMPARs is realized for additional activation/excitation of glutamate neurons during epileptiform activity. At the same time, activation of a specific population of GABAergic neurons does not inhibit but excites glutamatergic neurons: activation of CP-AMRARs (in GABA neurons) - increases GABA release, which via GABA(B)R activates Kv7 and hyperpolarizes and suppresses PDSs in other GABAergic neurons, thus leading to disinhibition of glutamatergic neurons.

## Data availability statement

The original contributions presented in the study are included in the article/supplementary material, further inquiries can be directed to the corresponding author.

## Ethics statement

The animal study was approved by all animal procedures were approved by the Bioethics Committee of the Institute of Cell Biophysics (ICB) and carried out according to Act708n (23 August 2010) of the Russian Federation National Ministry of Public Health, which states the rules of laboratory practice for the care and use of laboratory animals, and the Council Directive 2010/63 EU of the European Parliament on the protection of animals used for scientific purposes. ICB RAS Animal Facility provided the animals for experiments in accordance with the applications approved by the Commission on Biosafety and Bioethics of the Institute of Cell Biophysics (Permission No. 3, 12 April 2021; Permission No. 4, 17 June 2022). Pregnant rats were housed 2 to 3 in a cage in the ICB RAS animal facility with 12 h light–dark cycle and access to food and water *ad libitum*. The study was conducted in accordance with the local legislation and institutional requirements.

## Author contributions

VZ: Conceptualization, Project administration, Supervision, Writing – original draft, Writing – review & editing. IT: Formal analysis, Investigation, Methodology, Software, Visualization, Writing – original draft. AK: Data curation, Formal analysis, Methodology, Software, Visualization, Writing – review & editing. SG: Data curation, Formal analysis, Supervision, Validation, Visualization, Writing – review & editing. BK: Investigation, Visualization, Writing – review & editing. ST: Conceptualization, Funding acquisition, Project administration, Resources, Supervision, Validation, Writing – review & editing.
